# X-ray and MRI Correlation of Bone Tumors Using Histopathology As Gold Standard

**DOI:** 10.7759/cureus.27262

**Published:** 2022-07-25

**Authors:** Hina Azad, Aliya Ahmed, Ibtesam Zafar, Muzammil Rasheed Bhutta, Muhammad Ali Rabbani, Himesh Raj KC

**Affiliations:** 1 Radiology, Pakistan Institute of Medical Sciences, Islamabad, PAK; 2 Anatomy, CMH Multan Institute of Medical Sciences, Multan, PAK

**Keywords:** histopathology, soft tissue, mri, x-ray, bone tumor, imaging techniques

## Abstract

Introduction

Bone tumors are a common pathology of the musculoskeletal system being frequently encountered by clinicians. Radiological workup is a mainstay in the diagnostic workup of bone tumors. This study aimed to highlight the importance of plain radiography and MRI in the diagnosis of bone tumors keeping histopathology as a gold standard. It is a descriptive validation study conducted in the Radiology Department of Pakistan Institute of Medical Sciences Islamabad.

Methodology

The study included 92 patients with suspected bone lesions. After taking a complete history and receiving informed written consent. X-rays radiographs and magnetic resonance imaging were performed. X-ray radiograph and magnetic resonance imaging parameters were recorded and compared with the histopathology of lesions as a standard.

Results

The mean age of patients was 30.50 ± 8.95 years. Of 92 patients examined on X-ray, 51 (55.4%) had lytic lesions, 34 (37.0%) had sclerotic lesions, and seven (7.6 %) had mixed lesions. MRI revealed the location of the lesion. There were 25 (27.2%) bone lesions in diaphysis, 19 (20.7%) in metaphysis, nine (9.8%) at meta-diaphysis, and 32 (34.8 %) in the meta-epiphyseal region. These findings were later on confirmed with histopathological results.

Conclusion

MRI can differentiate soft-tissue components and periosteal reactions. An X-ray radiograph can provide information about bony matrix and calcifications within tumors. After analysis of imaging findings and histopathological results, it is concluded that these modalities can be used to diagnose bone tumors with high diagnostic accuracy.

## Introduction

Bone tumors are caused by either abnormal growth of bone-like tissue or soft tissue in the bones. Tumors are classified into benign or malignant and primary or secondary. Globally malignant bony lesions have two age incidences (between 10-20 years and 40-80 years). They also have gender predilection, with the incidence in males 1.5% more than in females [1]. A study conducted in the northern region of Pakistan found primary bone tumors accounted for 3.14% of all tumors [2].

The diagnostic evaluation of focal bone lesions involves information from the patient’s medical history (age, gender, malignancies, history of pain, injuries), examination of the lesion, radiographic examination of the margins, degree of cortical expansion, periosteal reaction, and previous imaging. Primary bone tumors are overshadowed by metastatic cancers such as carcinoma and hematologic malignancies. MRI helps differentiate malignant bony lesions from benign lesions. In small lesions, MRI is helpful as small lesions can be picked up on a diffusion-weighted image (DWI) sequence, on which diffusion restriction indicates more toward malignancy. MRI is also helpful in imaging to follow up after treatment [3].

Radiological assessment of bone lesions is difficult if specialized centers are not available. Conventional radiography plays a major role in bone tumor diagnosis. X-rays play their specific role in imaging diagnostic workups. Bone tumor aggressiveness is calculated by interpretation of its bony matrix on the radiograph. For local staging, MRI is considered the most effective method [4].

An accurate diagnosis requires the patient’s age, location of tumor, pattern of their destruction/margins, aggressiveness, growth speed, matrix formation, periosteal reaction (inside or outside), cortical involvement, size, number, and appearance on MR imaging [5,6,7]. Benign tumors are pathologies commonly encountered in daily practice and have various common characteristic appearances. Hence, it is essential for clinicians to evaluate their presentation, symptoms, and radiographic appearance to be safe from misdiagnosing.

Our study compared imaging features of X-rays and MRI in bone tumors with histopathological findings taken as the gold standard.

## Materials and methods

The study conducted was according to the Helsinki Declaration. It was a human study approved by the Ethical Review Board of Shaheed Zulfiqar Ali Bhutto Medical University (SZABMU)-approval F 1.1/2015/ERB/SZABMU/951. All adult participants provided written informed consent to participate in this study from July 2021 to October 2021. The inclusion criteria of this study are patients of all ages with mass lesions planned for an MRI scan. The exclusion criteria of this study are patients with a history of surgeries/known for any other malignancies or already taking chemotherapy.

The sample size was calculated as 92 cases with the WHO sample size calculator using the following parameters: sensitivity 92%; specificity 87%; expected prevalence 50%; precision level 8%, and confidence level of 95% [3].

After taking a complete history and informed written consent X-ray radiographs were conducted. Patients were positioned on the examination table; X-ray radiographs were taken with at least two projections anteroposterior and lateral views.

Once the imaging was completed, X-ray findings were interpreted by a consultant radiologist (at least three years of post-fellowship experience) and looked for the presence or absence of a bony lesion. X-ray findings were compared with MRI.

Data were recorded and analyzed through SPSS (Statistical Package for Social Sciences, IBM, Armonk, New York, USA) (V. 25). For continuous variables, mean ± SD were calculated, and categorical data were presented as frequency(percentage). Using a 2*2 table diagnostic accuracy, sensitivity, and specificity were calculated.

## Results

After acquiring informed consent, 92 participants were enrolled in the study. Their mean age was 31.3±9.61 years. There was a minimum age of 20 years and a maximum of 59 years. Of the 90 participants, 43 (46.7 %) were males, and 49 (53.3 %) were females. All 92 patients underwent X-rays and MRI, and results were recorded. Primary symptoms were recorded for all patients. Out of 92 patients, 42 (45.7 %) complained about pain, 24 (26.1 %) complained about swelling of joints, 15 (16.3 %) complained about weight loss, and 11 (12.0 %) complained about restricted movement, as shown in Table 1.

**Table 1:** Demographic characteristics of patients and primary symptoms noted

Features		N = 92
Age		31.3 ± 9.61
Gender	Male	43 (46.7%)
	Female	49 (53.3%)
Symptoms	Pain	42 (45.7%)
	Swelling	24 (26.1%)
	Weight loss	15 (16.3%)
	Restricted motion	11 (12.0%)
Other diseases	Diabetes	38 (41.3%)
	Hypertension	21 (22.8%)
	Arthritis	12 (13.0%)
	Others	12 (13.0%)
	Nil	9 (9.8%)

Diabetes was reported in 38 (41.3%) patients, hypertension in 21 (22.8%) patients, arthritis in 12 (13.1%) patients. All patients had their vital signs recorded; 70% of them had a mild or moderate fever. X-rays of 92 patients revealed 51 (55.4%) lytic lesions, 34 (37.0%) sclerosis, and only seven (7.6%) mixed lesions.

In 92 patients, 25 (27.2%) had bone lesions in diaphysis, 19 (20.7%) in metaphysis, nine (9.8%) in meta-diaphysis, and 32 (34.8 %) in meta-epiphyseal region as shown in Table 2. Similarly, 51 (54%) of them are lytic, 34 (37%) of them are sclerotic, and three (3.3%) of them are metastatic as described in Table 2.

**Table 2:** Vitals and findings of X-ray and MRI scans

Features	Cases (n = 92)	
Vitals	Fever	38.34 ± 1.32
	Heart rate	66.22 ± 12.7
	Respiratory rate	17.08 ± 3.86
	Blood pressure	117.2 / 86.5
Types of tumors	Primary	54 (58.7%)
	Secondary	34 (38%)
	Metastatic	3 (3.3%)
Type of lesions	Lytic lesion	51 (54%)
	Sclerotic lesion	34 (37%)
	Mixed lesion	7 (7.7%)
Site of lesions	Diaphysis	25 (27.2%)
	Metaphysis	19 (20.7%)
	Meta-diaphysis	9 (9.8%)
	Meta-epiphysis	34 (34.8%)
	Others	7 (7.6%)

**Figure 1:**
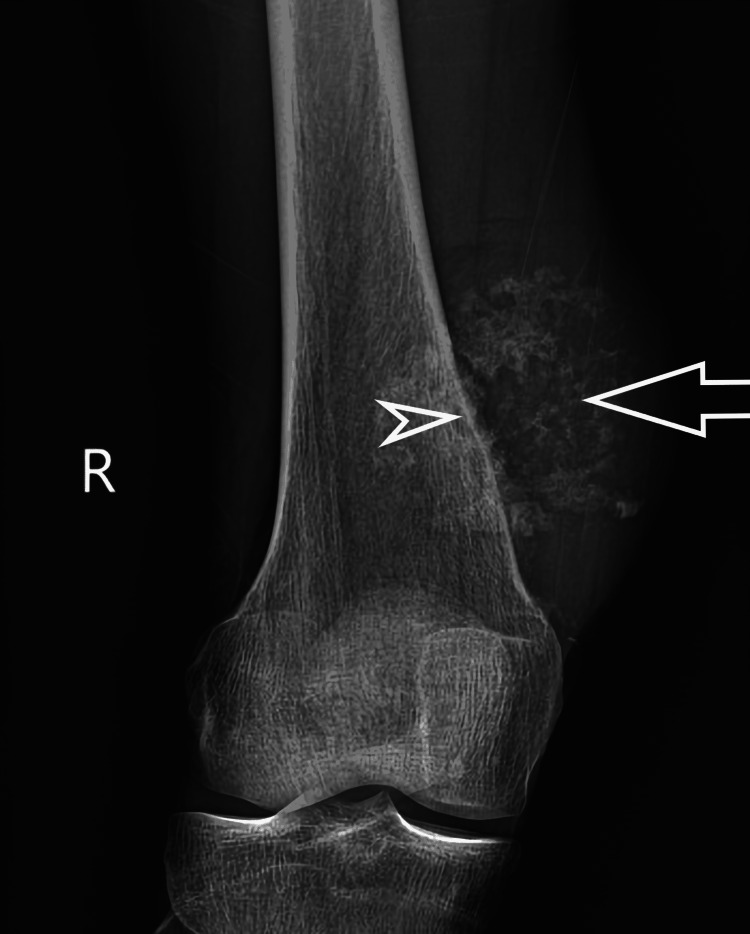
X-ray of a patient showing soft-tissue swelling at the medial aspect of distal femoral metaphysis containing amorphous calcifications (arrow) and subtle periosteal reaction of underlying bone (arrowhead). Histopathology revealed osteosarcoma.

In all 92 patients who had histopathology performed, 81 were found to have malignant bone tumors, and 11 were found benign. MRI and X-rays have almost equal diagnostic accuracy. Through X-ray imaging, osteoporosis, periosteal reaction, sclerotization of lesions, calcifications, and ossification of bone can be evaluated (Figure 1).

**Figure 2:**
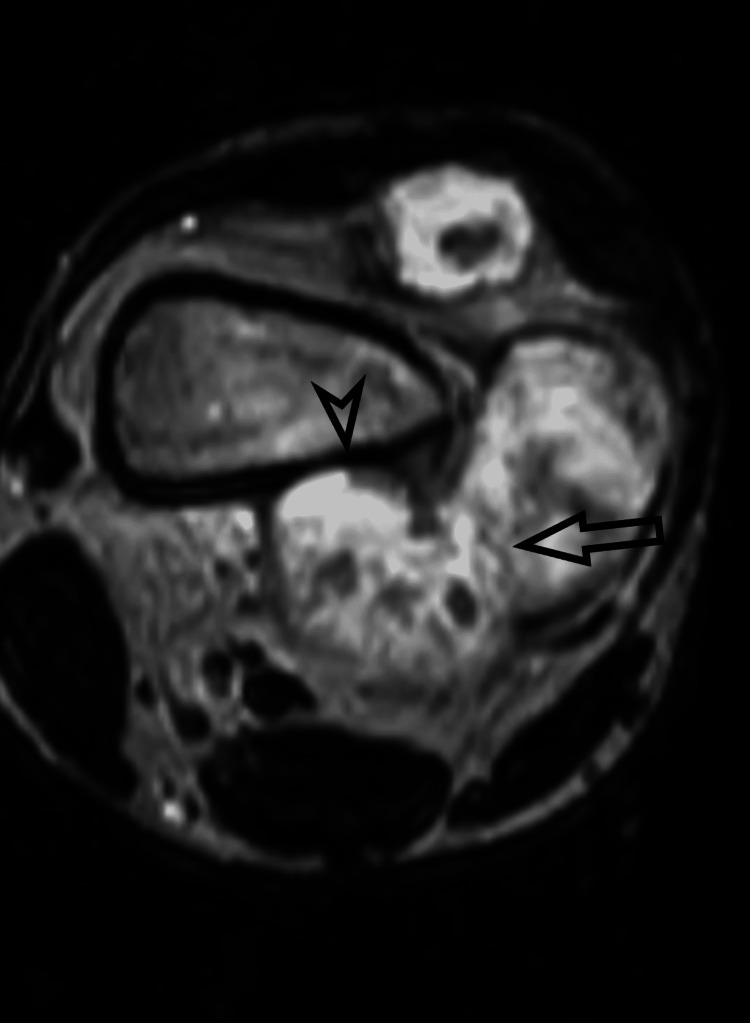
MRI scan of a patient illustrating the soft-tissue mass with its anatomical position and involvement of adjacent structures. Long arrow points to the soft-tissue component of tumor. Arrowhead marks the area of cortical breach. Histopathology revealed a parosteal osteosarcoma.

MRI allows evaluation of tissue properties, tumor extent, and reactive areas as shown in Figure 2.

Based on our results, the diagnostic accuracy of X-rays and MRI in the evaluation of bone tumors is as mentioned in Table 3.

**Table 3:** Diagnostic accuracy of X-ray and MRI for detecting bone tumors taking histopathology as gold standard

	Standard positive (n = 81)	Standard negative (n = 11)
X-ray		
Index test positives	76 (true positive)	5 (false positive)
Index test negatives	7 (false negative)	4 (true negative)
Sensitivity	91.56%	
Specificity	44.4%	
Positive predictive value	93.8%	
Negative predictive value	36.3%	
Diagnostic accuracy	86.95%	
MRI		
Index test positives	74 (true positive)	2 (false positive)
Index test negatives	6 (false negative)	5 (true negative)
Sensitivity	92.5%	
Specificity	71.42%	
Positive predictive value	97.3%	
Negative predictive value	54.5%	
Diagnostic accuracy	87.0%	

## Discussion

Our study evaluated the diagnostic accuracy of X-rays and MRI for bone tumors, histopathology was taken as gold standard for diagnosis. After taking histopathology as gold standard we found that MRI and X-ray have excellent diagnostic precision for bone tumors. Primarily malignant bone tumors affect long bones and the spine. This study aimed to highlight the importance of plain radiography and MRI in the diagnosis of bone tumors. There are some limitations to X-ray imaging in diagnosing any bone neoplasms, such as lesions in complex anatomy, bone marrow assessment, and soft-tissue resolution, which are crucial for staging. MRI is an excellent modality as it can diagnose bone tumors beforehand. It can also help to distinguish benign pathologies such as hemangiomas from the metastatic deposits as the presence of normal fatty marrow which appears in the form of a high signal on T1 recommends hemangioma, similarly cartilaginous cap of osteochondroma can also be well appreciated on MRI [8]. After analyzing all the radiological data and histopathological findings, we found out that X-ray radiograph has a diagnostic accuracy of 86.95% and MRI had a diagnostic accuracy of 87.0%, so both of them have high diagnostic accuracy, however, MRI is more precise for analyzing soft tissues and determining the extent of tumor growth.

A study conducted by Weber et al. revealed that patients suspected of malignant bone tumors have similar symptoms, including pain, swelling, and fever [9]. Of all symptoms, the pain was the most prevalent symptom in our study, with restricted movement being the least prevalent [10].

Jain et al. conducted a multimodality study to compare all imaging modalities used to diagnose malignant bone tumors. Among the aspects of tumor evaluation he discussed were degree of marrow involvement, involvement of overlying soft tissues, involvement of adjacent joints, and knowledge of skip lesions and metastases. CT, MRI, and other multimodal imaging aid in covering all these aspects [10]. Recent studies have shown that for the treatment of bony lesions, an early diagnosis is vital, as it is followed by biopsy and an early start of treatment helps to improve the patient’s condition, lessens metastatic spread, and increases life quality and life expectancy of the patient. MRI and X-rays are both useful for diagnosis. An MRI demonstrates local staging, soft-tissue involvement, and extent of the growth, as mentioned earlier. As Fritz et al. reported, we also found that MRI has high sensitivity and positive predictive value [6]. A study by Lange showed higher sensitivity, specificity and accuracy of MRI in comparison to other imaging modalities; MRI sensitivity, specificity, and accuracy were 90.5%, 80.1%, and 87.1%, however, in our study, sensitivity was 92%, and specificity was 71%. A study by Bhuyan and Bhuyan showed sensitivity and specificity of MRI in diagnosing bone tumors to be 100% and 98%, respectively [8,11].

Bony lesions after treatment show sclerosis in case of effective treatment and an increase in lytic activity of primary lesion in case of failure of treatment, both of these processes can be successfully evaluated on X-rays [12]. It is also evident that some of the bone tumor diagnosis is also established by correlating clinical history, plain radiograph, and histopathology [13].

In another study conducted by Lange, MRI proves to be the best modality considering spinal lesions and multiple myelomas and for assessing bone marrow infiltration. It is also helpful in early diagnoses and detection of the risk of vertebral fractures and in distinguishing between benign and malignant osteoporosis [14]. Magnetic resonance imaging of the spine and pelvis and positron emission computed tomography combined with X-ray radiograph of the skull have been proposed as the standard method in imaging workup of newly diagnosed cases of multiple myeloma [15]. Hemangiomas are most commonly encountered benign bony lesions and they are nowadays confidently diagnosed on the basis of MRI. MRI is also helpful in staging and monitoring bone tumors and along with DWI, it is used for the detection of pre- and post-treatment necrosis of bone tumors [16,17]. It is also the preferred imaging modality in skeletal metastasis workup of myxoid and round cell liposarcomas [18]. The collagenous matrix shows a hypointense signal on both T1/T2W, myxoid appears hypointense on T1 and hyperintense on T2, adipose appears hyper on both T1/T2 with intensity variation showing more intensity on T1 than T2. So, signal characteristics along with morphological and anatomical details are helpful in providing state of art patient care. There are a few limitations as this study was limited to a small number of study participants, and as it is an observational study, we may need a randomized controlled trial to have more comparable results. Although radiograph remains a mainstay for initial assessment and diagnostic workup, MRI helps for further characterization. So, both modality correlations should be stressed in the evaluation and imaging workup of bone tumors [19,20].

## Conclusions

Bone tumors can be diagnosed with high diagnostic accuracy using X-rays and MRIs, which are reliable and noninvasive imaging methods. On X-rays by evaluating bone and soft-tissue calcification, and on MRI local invasion, soft-tissue characterization, perilesional changes, and diffusion restriction rule out malignancy and minimize errors and need for fine-needle aspiration cytology (FNAC) or biopsy with high diagnostic accuracy.
